# Does Continuous Renal Replacement Therapy with oXiris in Septic Shock Have Any Positive Impact? Single-Centre Experience with oXiris Therapy in Septic Shock Patients

**DOI:** 10.3390/jcm13247527

**Published:** 2024-12-11

**Authors:** Wojciech Mielnicki, Agnieszka Dyla, Marta Zając, Natalia Rokicka-Demitraszek, Jacek Smereka

**Affiliations:** 1 Anestesiology and Intensive Care Ward, Olawa District Hospital, 55-200 Olawa, Poland; w.mielnicki@zozolawa.wroc.pl (W.M.); dylusia@wp.pl (A.D.); zajac_19@op.pl (M.Z.); nrokicka-demitraszek@wp.pl (N.R.-D.); 2 Department of Emergency Medical Service, Wroclaw Medical University, 51-616 Wroclaw, Poland

**Keywords:** septic shock, CRRT, CVVHDF, oXiris hemofilter

## Abstract

**Background**: Renal replacement therapy with an oXiris hemofilter may be helpful for patients with acute kidney injury in conjunction with sepsis and septic shock. The aim of this study was to assess the impact of an oXiris membrane on septic shock patients. **Methods**: All renal replacement therapies with oXiris (Baxter, Deerfield, IL, USA) performed between January 2018 and August 2021 were retrospectively analyzed. CRRT was initiated in continuous venovenous hemodiafiltration (CVVHDF) mode using Prismaflex System (Baxter). Demographic data, starting point of infection, source control, etiology, and course of treatment were analyzed. **Results**: A total of 32 patients were included in the study. Most patients treated with oXiris had acute kidney injury (AKI) and required CRRT. One patient had KDIGO 1 AKI (3.1%), three patients (9.4%) had KDIGO 2 AKI, and 28 patients (87.5%) had KDIGO 3 AKI. A statistically significant decrease in vasopressin dosage was required to achieve adequate MAP after 24 and 72 h, and a statistically significant decrease in norepinephrine dosage after 72 h was observed, with no SOFA score change on days 2 and 3. Procalcitonin and lactate levels did not change after 24 and 72 h. No beneficial effect on mortality was observed. **Conclusions**: Treatment with an oXiris membrane can positively impact vasopressors’ requirement but not influence SOFA score, procalcitonin or lactate levels, or mortality in septic shock patients.

## 1. Introduction

Sepsis is among the leading causes of morbidity and mortality in critically ill patients. It is estimated that almost 50 million people in the world are affected with sepsis every year, leading to 11 million deaths, which accounts for almost 20% of all global deaths [1,2,3]. The incidence of sepsis, treatment, and prognosis depends on the geographic area, comorbidities, population ageing, treatment-resistant microorganisms, demographic factors, and health care expenditures [4]. In countries with limited resources, there are real difficulties in diagnosing sepsis and starting effective treatment promptly. This includes the availability of intensive care and the ability to implement advanced treatment. Due to its heterogenicity, it is difficult to generate reproducible data on mortality in sepsis and septic shock, with ranges between 15% and 56% being reported in the literature [2,5,6]. Infection leading to sepsis and septic shock triggers dysregulated immune responses in the body. This cytokine storm is responsible for organ failure [6,7].

One of the critical factors of a negative outcome in septic shock is acute kidney injury, which can be present in up to 50% of patients [8]. Some patients with AKI require renal replacement therapy to remove toxic metabolites and excessive water. A benefit of CRRT is blood purification from endotoxins and cytokines that are present during septic shock [9]. Apart from standard dialysis filters, some membranes are equipped with special adsorption capabilities. An oXiris hemofilter is a high-permeability polyacrylonitrile (AN69)-based membrane, enriched with a positively charged polyethyleneimine surface, which adsorbs negatively charged endotoxins [10]. It is the only hemofilter to simultaneously provide renal replacement therapy, remove endotoxin molecules, and adsorb cytokines [11,12].

By removing endotoxins and adsorbing cytokines, one might expect a decreased inflammatory state, a reduction in vasoplegia, capillary leaks and, eventually, organ improvement. However, the clinical effectiveness of an oXiris membrane is still being analyzed in the published literature [11]. We present our single-centre experience with an oXiris hemofilter and its impact on patients with septic shock. The aim of the study was to evaluate the influence of an oXiris filter on the dose of vasopressors, SOFA score, procalcitonin, WBC, lactate levels, and mortality.

## 2. Materials and Methods

This was a retrospective single-centre, observational study conducted in the Department of Anesthesiology and Intensive Care of the District Hospital in Olawa, Poland. This six-bed Intensive Care Unit treats around 200 medical and surgical patients annually.

Inclusion criteria: Patients in septic shock with a starting point in the abdomen or urinary tract (due to the high risk of Gram-negative etiology) and patients who required high doses of catecholamines (norepinephrine ≥ 0.2 ug/kg/min, argipressin ≥ 0.02 I.U./min). Exclusion criteria: COVID-19, failure to control the source of infection, and concomitant diseases all disqualified patients from expanding ICU therapy.

All renal replacement therapies with an oXiris hemofilter performed between January 2018 and August 2021 were analyzed. Demographic data, indications for oXiris and renal replacement therapies, and the starting point of infection, source control, etiology, and the course of treatment in septic shock patients were analyzed.

The Ethical Committee of Wroclaw Medical University approved this study (KB 977/2022).

CRRT was always initiated in continuous venovenous hemodiafiltration (CVVHDF) mode using the Prismaflex System (Baxter, Deerfield, IL, USA). An effective CRRT dose was between 20 and 35 mL/kg/h. We used regional anticoagulation with citrate in all patients, and an oXiris hemofilter was used for 72 h. The decision regarding the continuation of CRRT was individualized and, if necessary, an ST150 hemofilter was used. The mode of CRRT or the effective dose prescription range was not changed during the treatment.

Assessing the effectiveness of an oXiris membrane in the treatment of severe septic shock, we analyzed the mean arterial blood pressure (MAP) and sequential organ failure assessment (SOFA) scores as indicators of disease severity during treatment while also examining catecholamine doses (norepinephrine and vasopressin), and laboratory parameters (WBC, procalcitonin, blood lactate). All the parameters were analyzed before oXiris treatment after 24 and 72 h.

Statistical analysis: A normal distribution data analysis was performed using a paired t-student test. Non-parametric data were compared using the Wilcoxon test. Data distribution was evaluated with the Shapiro–Wilk test. Tests were conducted using the Python environment (ver.3.11). We used *p* < 0.05 as the level of statistical significance.

## 3. Results

Between January 2018 and August 2021, we performed 32 CRRT therapies with oXiris hemofilter patients aged 37 to 84 (median age—69). Upon admission to the ICU, their combined SOFA score was calculated as 7–16 (median 10), while their SAPS III was 46–93 (median 70) and the corresponding mortality risk was 16–93% (median 69%). Demographic data and the patients’ conditions are presented in Table 1.

Between January 2018 and August 2021, we performed 32 CRRT therapies with oXiris hemofilters. There were 32 patients treated with an oXiris filter aged from 37 to 84 (median age–69 years old). Upon admission to the ICU, their combined SOFA score was calculated as 7–16 (median 10) and their SAPS III was calculated as 46–93 (median 70), with a corresponding mortality risk of 16–93% (median 69%). Demographic data and the patients’ conditions are presented in Table 1.

CRRT with an oXiris hemofilter was initiated between 6 h and 62 days after ICU admission (median 30 h). The main reason for ICU admission was septic shock, and, in all 27 patients with this diagnosis, we started CRRT with oXiris in the first 72 h of ICU stay. In five patients, septic complications developed in the late phase of ICU stay; thus, oXiris therapy was also initiated later (6 days–62 days).

In all patients, refractory septic shock was the main indication for CRRT with an oXiris membrane. Table 2 presents the site of infection and its etiology.

Most patients treated with oXiris had an acute kidney injury (AKI) that required CRRT. Only one patient (3.1%) had KDIGO 1 AKI, while three patients (9.4%) had KDIGO 2 AKI and 28 patients (87.5%) had KDIGO 3 AKI.

**Figure 1:**
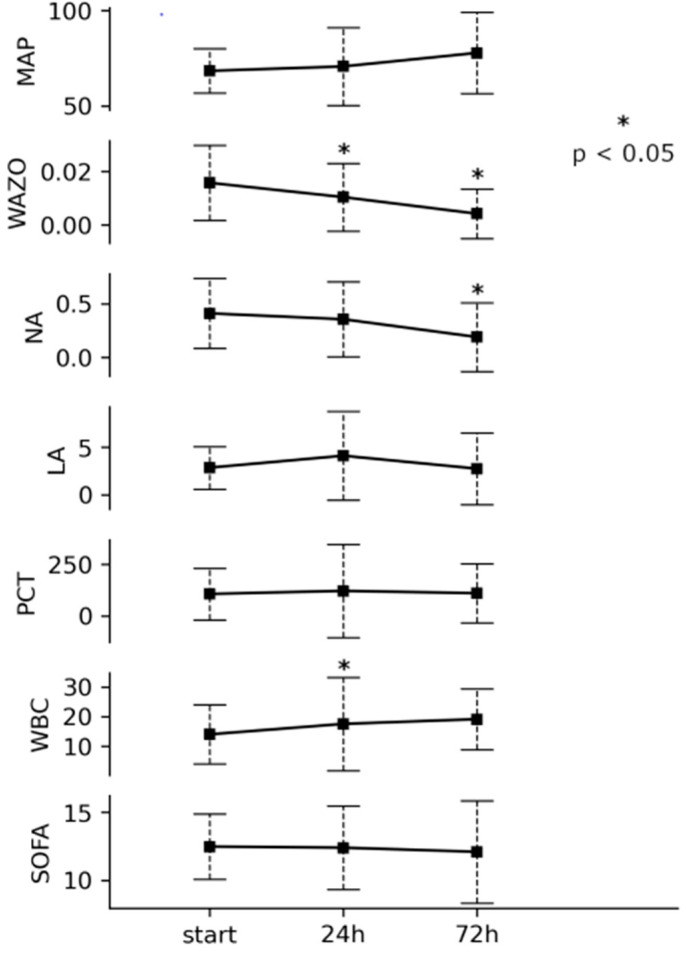
Changes in selected parameters after 24 h and 72 h of oXiris treatment.

During treatment with an oXiris hemofilter, we observed a statistically significant decrease in the vasopressin dose required to achieve adequate MAP after 24 and 72 h *p* < 0.05) and a statistically significant decrease in the norepinephrine dose after 72 h (*p* < 0.05). However, it did not change the SOFA score on days 2 and 3 of the ICU stay (Figure 1). 

Assessing selected laboratory parameters, we did not observe a significant decrease in procalcitonin levels after 24 and 72 h. WBC levels increased transiently after 24 h (*p* < 0.05) and lactate levels did not change during treatment with oXiris.

Clinical improvement was achieved in nine patients (28%) discharged from the ICU after 6 to 51 days (median 13 days). The remaining 23 patients (72%) died after 1 to 74 days (median 6 days) of ICU stay.

## 4. Discussion

Despite advanced treatment of severe sepsis and septic shock (early antibiotics, surgical source control), the mortality rate remains high, being significantly high when AKI complicates the clinical picture [8]. Inflammatory response initiated by severe infection can lead to multiorgan failure and death due to excessive production of cytokines [12]. Blood purification techniques to remove endotoxins and cytokines [13,14,15] are an interesting complementary treatment for severe sepsis and septic shock. The adsorbing membrane oXiris is a modified AN69 surface-treated hemofilter that can remove both endotoxins and cytokines. The removal is potentiated by increased polyethene imine surface coating and increased immobilized heparin [16]. Because this entity is so versatile, finding the optimal balance between pro- and anti-inflammatory responses makes treatment challenging.

We present our single-centre experience with an oXiris hemofilter in refractory septic shock in 32 ICU patients. Most of the patients (87.5%) had KDIGO 3 AKI before initiation of CRRT, had high levels of norepinephrine and vasopressin infusions (SOFA 4 points for circulation), and had very high SAPS III upon admission (median 70 points, mortality risk 69%). We hypothesized that early initiation of CRRT with an oXiris hemofilter in severely ill septic patients may improve their clinical condition, decrease inflammatory parameters, and reduce mortality.

Our results show a statistically significant reduction in norepinephrine and vasopressin infusions. A similar effect on norepinephrine doses was shown by Turani et al. [16] in the analysis of medical records of 60 patients treated for septic shock with an oXiris hemofilter between April 2011 and December 2018. Eighty-five percent of patients included in the analysis had KDIGO 3 AKI. The same hemodynamic effect of oXiris was documented in a prospective, randomized crossover, double-masked study of 16 patients between February 2016 and February 2018. These patients had KDIGO 3 AKI and confirmed or suspected Gram-negative septic shock (endotoxin > 0.03 EU/mL). Patients treated with oXiris required lower doses of norepinephrine to sustain mean arterial pressure [17]. Another study showing the benefit of oXiris on norepinephrine infusion was conducted by Schwindenhammer et al. [10]. Thirty-one patients were included in the study, and the patients who benefited most were the ones with abdominal sepsis and Gram-negative bacilli etiology. Lui et al. compared an oXiris hemofilter with the conventional hemofilter ST 100. Norepinephrine doses were significantly lower in the oXiris group compared to the control group 48 h after CRRT [18]. The positive hemodynamic effect was also observed by Zhou et. in the retrospective analysis of 90 patients with sepsis/septic shock who underwent at least one oXiris-treatment [19].

Feng et al. presented a randomized controlled trial that enrolled 16 surgical septic shock patients with AKI admitted to the ICU. They observed that, in the oXiris group, lactate levels decreased and the norepinephrine infusion rate decreased more compared to the standard filter AN69-ST group [20].

Despite the reduction in catecholamines, we did not observe a change in SOFA score on day 2 or 3, which differs from observations conducted by Sham et al. [21]. In their retrospective case series on septic patients with AKI, they found that the use of oXiris in CVVH mode led to a decrease in SOFA scores after 48 compared to historical controls. Turani et al. presented the same effect on the SOFA score [16]. In the Lui et al. study, the SOFA score was also significantly lower in the oXiris group compared to the control group 48 h after CRRT [18]. Zhou et al. concluded that the SOFA score was significantly improved at 12 h and 24 h after treatment with an oXiris filter [19]. SOFA scores depend on several factors, and different results have been obtained in the various studies cited. However, this may be due to the heterogeneity of the study groups and the relatively small number of study groups.

Our results align with the observations performed by Schwindenhammer et al. [10]. They did not observe significant improvement in SOFA scores 24 or 48 h after initiating CRRT with oXiris. The SOFA score might not change due to the very high initial dose of norepinephrine, which did not decrease below the 0.01 mcg/kg/min necessary to change the SOFA score. The use of vasopressin is not included in the SOFA score, so any decrease in the dose has no potential impact on SOFA calculation.

Regarding laboratory parameters, we did not observe a significant decrease in procalcitonin and lactate levels; however, WBC increased transiently in 24 h. Turani et al. observed the beneficial effect of oXiris therapy on procalcitonin levels [16]. Liu et al. concluded that the WBC, high-sensitivity C-reactive protein, serum lactic acid, and procalcitonin levels were significantly lower in the oXiris group compared to the control group [18]. In the Zhou et al. study, inflammatory biomarker levels were significantly lower at 12 h and 24 h after treatment [19]. Procalcitonin was an interesting marker because our laboratory had no technical capabilities to measure endotoxin or cytokine levels. By starting oXiris therapy, we might observe a reduction in procalcitonin and WBC as markers of treatment effectiveness. Our results were disappointing in this regard.

Our patients treated with an oXiris hemofilter had very high mortality, reaching 72%. This result was consistent with the SAPS III mortality risk calculated upon admission. Data published in the literature are conflicting on the topic of mortality. Schwindenhammer et al. [10] found lower mortality in patients with oXiris, whereas Shum et al. [21] did not observe any change in mortality. Guan et al. published a retrospective observational study comparing the efficacy of adsorbing filter oXiris in treating septic shock acute kidney injury. The early mortality at 7 and 14 days was significantly lower in the oXiris group compared with the ST150 group (7 days: 47.1 vs. 74.2%, *p* = 0.007; 14 days: 58.5 vs. 80.3%, *p* = 0.005), but the difference was not significant in regard to 90-day mortality [22]. Xie et al. presented a study on 30 patients with septic shock treated with oXiris-CVVH, and 46 patients with septic shock were treated with AN69 filter-CVVH. The 28-day mortality in the control group is higher than in the treatment group (73.3% vs. 47.3%, *p* < 0.001; median survival time: 10 vs. ≥28 days) [23]. By starting oXiris therapy with our severely ill septic patients, we expected its beneficial and complementary role in the treatment process.

A meta-analysis published by Siew et al. showed that using an oXiris membrane was associated with diminished overall mortality, norepinephrine infusion, CRP, IL-6, and lactate levels, together with improved organ functions [24]. Their meta-analysis pointed out the low quality of the studies analyzed and the need for further RCTs. Similarly, a meta-analysis published by Wang et al. showed that patients with sepsis treated with an oXiris filter significantly reduced 28-day mortality, as well as ICU hospitalization time, compared to patients treated with standard filters [25]. There was also a reduction in SOFA scores, norepinephrine infusion, and lactate and IL-6 levels. Unfortunately, they did not observe differences in the 90-day mortality, hospital mortality, and length of hospital stay.

Limitations. Data presented in this article must be evaluated in the context of certain limitations. This article is a retrospective analysis of the case series. It was impossible to compare it to any control group because oXiris therapy was initiated as a standard protocol for septic shock requiring CRRT starting in 2018. The aim was to use its potential for renal replacement therapy, the removal of endotoxin molecules, and the adsorption of cytokines. Although the number of patients included in the study is relatively low, it adds to the data published on oXiris therapy in ICU patients. Patients included in the study were severely ill, and the decision to implement an oXiris membrane was often made during disease progression. We cannot rule out the irreversibility of septic shock at the moment of treatment initiation, which eventually translated to poor outcomes. Despite filter effectiveness, we also cannot rule out the underdosing of antibiotics during CRRT with oXiris. Low antibiotic concentration during CRRT in septic patients happens [26] and might lead to treatment failure. We did not use sophisticated laboratory methods to evaluate the effectiveness of oXiris membranes (endotoxins, cytokines). We concentrated on simple parameters available in almost every hospital. We used our membrane for 72 h, which might have decreased the filter’s adsorbing capabilities. Tan et al. changed their filter every 12 h for three consecutive days [27]. Finally, our observations in the first 72 h showed no change in the SOFA score. We might have observed a change after that period if we had continued the analysis, but it was not our priority. An important element that needs to be clarified is the timing of including oXiris filters in therapy. Prompt initiation of this type of filter has a rationale based on the pathophysiology of septic shock. More rapid initiation of this type of therapy may affect overall patient outcomes; in any case, further studies with a more homogeneous timing of inclusion in terms of this type of therapy are needed to allow for analyses of more homogeneous groups.

## 5. Conclusions

Treatment with an oXiris membrane can positively impact vasopressors’ requirements but not influence SOFA scores, procalcitonin or lactate levels, or mortality in septic shock patients.

## Figures and Tables

**Table 1:** Demographic data and patients’ conditions upon admission to the Intensive Care Unit (ICU).

Number of patients	32
Age (years, SD)	37–84 (69)
Sex (M:F)	14:18
Charlson score (SD)	0–18 (16)
SAPS III (upon admission/mortality risk)	46–93 (70)/16–93% (69%)
SOFA (upon admission)	7–16 (10)

**Table 2:** Infection characteristics.

Site of Infection	
Abdominal	23 (71.9%)
laparotomy	20 (87%)
Intestinal perforation	11
Ischemia	3
Mechanical ileus	3
Cholecystitis	3
Pancreatitis	1
Pneumonia	7 (9.4%)
Urosepsis	2 (6.3%)
Etiology	
Identified pathogen	28 (87.5%)
Gram-negative bacilli	20
Enterococci	6
Positive blood cultures	16 (50%)

## Data Availability

The data used in the analysis are available from the corresponding author upon request.
